# Earmarking for global health: benefits and perils of the World Bank’s trust fund model

**DOI:** 10.1136/bmj.j3394

**Published:** 2017-08-31

**Authors:** Janelle Winters, Devi Sridhar

**Affiliations:** Medical School, Edinburgh University, Edinburgh, UK

## Abstract

In the third article in this series, **Janelle Winters** and **Devi Sridhar** review different types of trust funds and how they fit within the bank’s lending mechanism, and discuss the major benefits and risks of the bank’s use of the trust fund model for health

Over the past 50 years, the World Bank has increasingly relied on resources contributed voluntarily from donors and held separately from its core budget to support projects and activities, particularly for global health.^1^ These resources are known as trust funds. In the case of the bank, these trust funds are synonymous with earmarked, extra-budgetary, and “multi-bi” aid (bilateral aid channelled through multilateral institutions).^2^ ^3^ The absolute number and relative proportion of bank assets held in trust has skyrocketed since the early 1990s. In 2011, the bank was trustee to roughly half of the trust funds for official development assistance (ODA) worldwide,^4^ ^5^ and in 2012-13 about 200 donors contributed $3.7bn to more than 1000 World Bank Group trust funds.^6^

## Trust fund governance at the bank

The World Bank Group channels voluntary grants from donors in three major ways: through IBRD (International Bank for Reconstruction and Development) and IDA (International Development Association) trust funds, financial intermediary funds, and IFC (International Finance Corporation) trust funds. In this article we focus primarily on the World Bank’s IBRD and IDA trust funds and financial intermediary funds (table 1, see the first paper of this series for more on the World Bank Group’s structure).^7^ At IBRD/IDA, trust funds are classified as bank or recipient executed, depending on the bank’s management role. Bank executed trust funds are implemented directly by the bank.^8^ ^9^ They typically fund technical support for IBRD/IDA country projects, provide seed funding for pilot projects, or contribute to the bank’s knowledge agenda. For recipient executed trust funds, the bank hands over implementation of the project to a third party, such as a country’s Ministry of Health or a non-governmental organisation.^4^ ^5^ ^8^ ^9^ These trust funds generally co-finance IBRD/IDA lending operations, finance stand alone projects, or support debt servicing operations.^8^ ^9^ Some recipient and bank executed trust funds share governance structures called “facilities,”^4^ ^10^ which are designed to increase coordination. To reduce fragmentation and better recover costs from managing IBRD/IDA trust funds, the bank recently announced a policy to replace recipient executed trust funds with “hybrid funds,” which will have bank executed components.^11^

**Table 1 tbl1:** Categories of World Bank trust funds and their general characteristics

	General purpose	Bank role	Donors	Geographical focus	Accountability	Fees
IBRD/IDA Bank Executed Trust Fund (BETF)	Project analytical and advisory services; project administration; bank’s knowledge agenda	Implementation. Funds support bank’s work programme	Single donor (majority); multi-donor	Country projects (majority)	Subject to bank administrative (not operational) policies; bank prepares terms of reference, procures goods/services, makes payments, and submits financial and programme reports to donors	Indirect rate of 17% of personnel costs charged on disbursement
IBRD/IDA Recipient Executed Trust Fund (RETF)	Co-financing IBRD/IDA operations; financing technical assistance	Operational. Funds pass to third party for implementation; bank appraises or supervises funded activities	Single donor (majority); multi-donor	Country projects (majority)	Subject to bank operational policies; recipients (implementers) submit progress and audited finance reports to bank. Some RETFs (>$5m or co-financing) can instead follow only administrative policies	For new “hybrid” funds, indirect rate of 17% for BETF portion charged on disbursement; scaled fees for RETF portion charged at contribution: 5% fee on first $50m, 4% fee on next $450m, 3% fee on next $500m, 2% fee on further contributions
Financial intermediary fund (FIF)	Providing complex trustee services for funds transferred to multiple implementing agencies	Financial trustee. Customised administrative, financial, or operational services	Multi-donor (vast majority)	Regional and global programmes (vast majority)	Case by case operational, administrative, and financial policies. Standard bank policies do not apply	Case by case. Cost recovery policy generally applies

Financial intermediary funds are more flexible and complex financing mechanisms that—with rare exception—support global or regional partnerships. For this type of trust fund, the bank negotiates a customised agreement with implementing agencies, which generally makes the bank trustee of funds from multiple donors.^4^ ^9^ ^12^ In some cases, the bank’s role is restricted to holding, receiving, and transferring commitments on behalf of legally independent implementing agencies (for example, the Global Fund to Fight AIDS, Tuberculosis and Malaria), while in others the bank is both trustee of and major donor to the fund (for example, the African Programme for Onchocerciasis Control). Finally, IFC trust funds typically finance advisory services to businesses and governments, as part of its institutional mandate to promote private sector investment in developing countries. An example is the $1bn Health in Africa Initiative, which is designed to channel funds for private health facilities and healthcare delivery and has received donor support through small trust funds and a large private equity fund.^13^ ^14^

Collectively, trust funds allow the bank to increase its income and role in global activities. At the end of 2016, the bank served as steward to about $11bn in trust fund resources at IBRD, IDA, and IFC, and held $23bn in financial intermediary funds.^15^ These resources finance a considerable proportion of the bank’s staff and operations, including over 60% of all global partnerships and nearly two thirds of the bank’s advisory and analytics work.^15^ ^16^ In 2013, trust fund revenue was almost as substantial as that of the entire IDA.^16^

## Trust funds for health: a 2012-13 snapshot

How much of this trust fund portfolio is dedicated to health projects? According to statistics reported by the bank in 2013, about half of the cumulative commitments to financial intermediary funds to date was for the health sector (fig 1). In 2012-13 alone, cash contributions to financial intermediary funds for the health sector totalled around $3.9bn, or 37% of the total cash contributions to bank trust funds.^9^ The same year, recipient executed trust funds handed out about $430m to the health and social services sector, which represents around 4% of the total disbursements from bank trust funds (fig 2).^9^ ^17^ Data are not available on bank executed trust fund financing for the health sector, but their relative contribution to the health sector is small as bank executed trust funds for all sectors accounted for just 6% of all trust fund disbursements in 2012-13.^9^ To put these numbers in perspective, IBRD/IDA lending for core health and social services projects was just over $5bn in 2012-13.^18^

**Figure f1:**
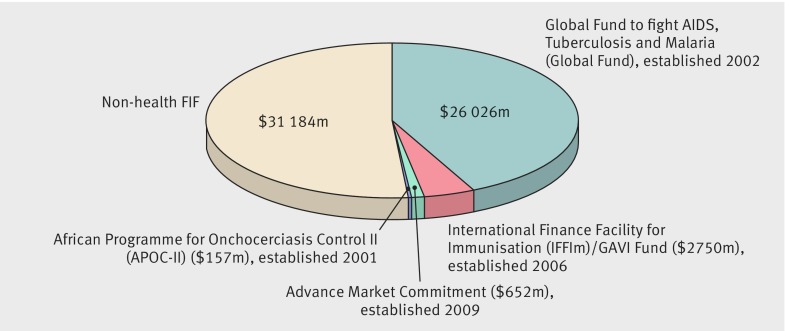
**Fig 1** As of June 2013, the health sector represented 48.7% of active financial intermediary fund (FIF) cumulative funding. Based on data from World Bank annual trust fund report (2013)

**Figure f2:**
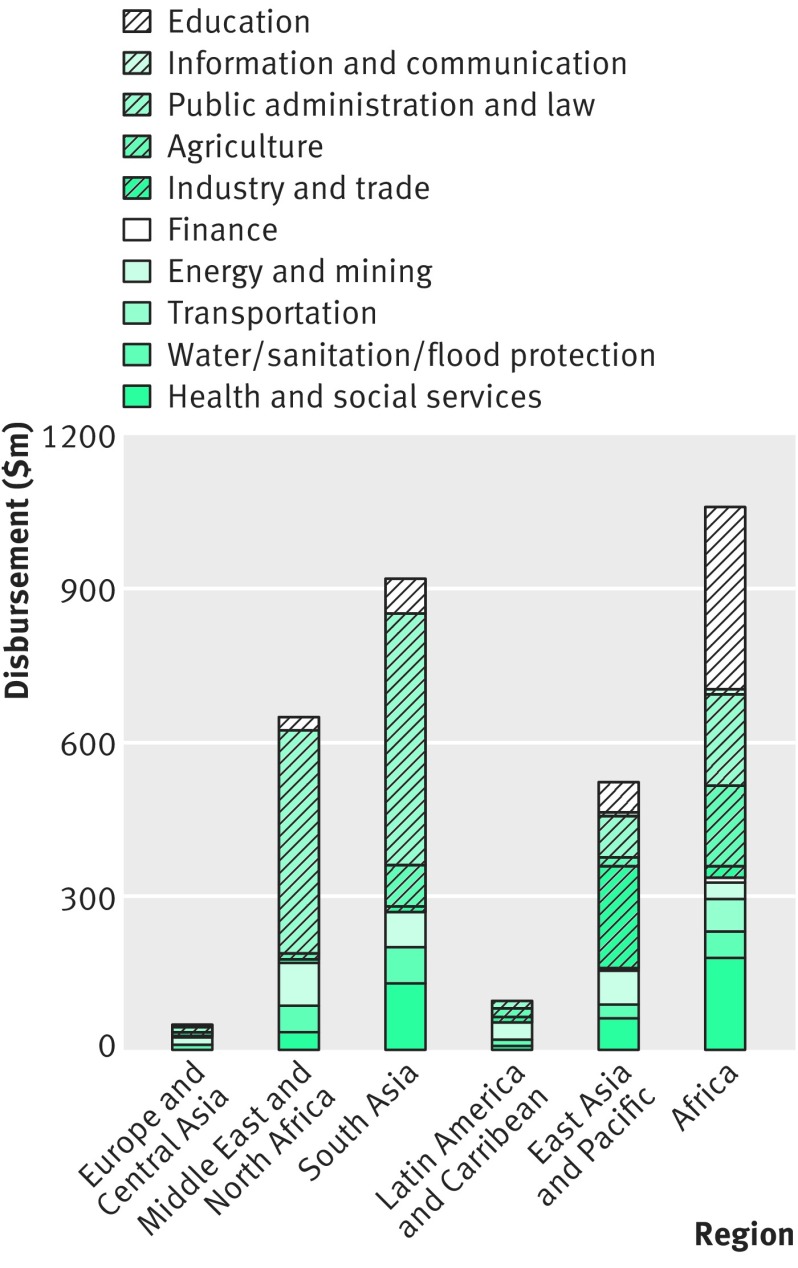
**Fig 2** Africa and South Asia received the largest disbursements of recipient executed trust funds for health in 2012-13. Based on data from World Bank annual trust fund report (2013)

## Benefits of the trust fund model for health

What has driven the bank to increasingly turn to trust funds to finance health projects? Firstly, the flexibility of trust funds allows the bank to raise funds from a diverse group of donors for priority countries, while retaining the bank’s trusted financial management services.^6^ ^16^ ^17^ Core IBRD/IDA programmes can accept contributions only from governments, but trust funds can accept funding from the private sector. This is especially important for vertical (disease specific) funds: corporations like Exxon Mobil and pharmaceutical companies have contributed to health trust funds, and the Bill and Melinda Gates Foundation was the second largest donor to the recent replenishment of Gavi, the Vaccine Alliance.^6^ ^19^ New financing mechanisms for many vertical funds also entice donors by allowing them to make programme and budgetary decisions. The Global Fund and Gavi, for example, have their own legal charters and a board of directors on which for-profit private sector representatives have voting power.^20^ Finally, trust funds can channel funding to countries that are not members of the bank or do not choose to invest in global public goods.^6^ For instance, the Avian and Human Influenza Facility raised $126m for avian influenza surveillance and control in 2006-13 and allocated some of this funding to “weak link” countries that were not prioritising influenza control interventions.^21^

Secondly, rapid agreement and disbursement of funds allows trust funds to channel the requests of specific donors in the context of specific international events or initiatives.^5^ ^6^ Donors have explained that they earmark aid because it allows them to respond more quickly to emerging challenges.^6^ Most trust funds are able to disburse funds more rapidly^22^ than core IBRD/IDA funding mechanisms because they sidestep traditional bank administrative and operational processes. For example, unlike in core lending, the bank’s board of executive directors usually are not required to approve trust fund proposals.^23^ Such ability to harness political momentum has been crucial to start up many global health programmes targeting infectious diseases.^4^ ^19^ ^20^

Thirdly, the narrowly defined goals and measurability of outcomes of trust funded projects make them attractive to donors.^6^ Trust funds for communicable disease control have increasingly dominated the bank’s trust fund portfolio over the past 15 years (figs 1 and 2[Fig f1 f2]).^5^ ^12^ Some private donors to these funds—particularly the Gates Foundation—have strong preferences for financing technological and disease specific interventions.^24^ ^25^ The outcomes of these grants are usually measured by simple metrics, like the number of bed nets, vaccines, or drug tablets distributed in specific countries.^26^ ^27^ Furthermore, because public and private donors to trust funds are often able to earmark their commitments to specific regions or activities, they are able to trace what their aid is buying at the country level.^6^

Finally, trust funds permit the bank and donors to support innovative regional and global projects that do not fit with the IBRD/IDA country focused allocation system. The bank has highlighted how trust funds allow it to expand its global partnerships for global public goods, emergency response, novel focuses (like gender), and, crucially, the control of communicable diseases.^4^ ^10^ ^12^ ^15^ Several health trust funds have also allowed the bank to fundraise in new ways or to pilot new financing mechanisms. For instance, the bank has used trust funds to incentivise IDA loans for maternal and child health (see paper 4 of this series on the Global Financing Facility^28^), to encourage donors to buy down IDA loans for countries investing in polio control,^29^ ^30^ and to provide Wall Street based insurance against future global pandemics (see paper 5 of this series on the Pandemic Emergency Financing Facility^31^). Similarly, trust funds supporting health results based financing have enabled the bank to pilot performance based financing at a village level before deciding whether to apply this strategy nationwide.^4^

## Risks of the trust fund model for health

Several costs, however, emerge from pursuing this model of investment. One major concern is that the bank has become vulnerable to “Trojan multilateralism” or the increased influence of small groups of donors on its health programming.^22^ ^32^ Indeed, major donors have reported that trust funds are a mechanism for bypassing existing allocation systems and influencing the bank’s priorities.^6^ This could tilt health funding toward vertical interventions and away from health priorities in the recipient country.^22^ ^33^ ^34^ For instance, the bank’s Independent Evaluation Group found that the polio buy down programme focused exclusively on providing polio vaccines and not wider health or social services infrastructure^4^ and that delivery of vertical funds sometimes overburdened weak national health systems.^16^ Additionally, two key measures that allow IDA to provide performance based allocation for its core projects—the country policy and institutional assessment and worldwide governance indicators—do not apply to trust funds, which further raises the risk that trust funds might not fit the needs of low income countries.^3^ ^4^

A second risk is that trust funds erode capacity of core health, nutrition, and population staff and weaken accountability mechanisms at the bank. While trust funds do not tend to increase the total amount of funding that sovereign donors give to the bank, their separate approval and allocation processes might increase transaction costs for the bank and recipient countries.^2^ ^16^ This can erode capacity of bank staff to supervise other country based health projects^16^ and explains why the bank recently began to charge higher, more consistent fees for trust funds.^17^ ^35^ The bank’s cost recovery framework (charging higher/consistent fees for trust funds) was initiated to prevent capacity erosion—if it charges consistent overhead costs for each trust fund, it can use this income to hire more staff or pay HNP staff directly for their work on trust funds.

There is an especially high risk that trust funds that finance global health partnership programmes will lack oversight and accountability. The bank does not have a central unit to oversee its participation in global partnerships,^16^ and the financial intermediary funds that typically fund these partnerships are not covered under the bank’s standard fiduciary, operational, or administrative policies.^36^ Environmental controls, overhead fees, and the public’s access to information on financial intermediary funds (FIFs) are therefore variable and not guaranteed.^4^ ^10^ ^37^ ^38^ Bank executed trust funds also came under fire in 2015, when it was made clear that they do not fall under the mandate of the bank’s inspection panel—the body that countries can turn to if they feel that safeguards have been compromised by a bank project.^39^ ^40^

Finally, the bank’s recent claim that it is a “champion for transparency and accountability”^15^ in its use of trust funds contrasts with the reality of trust fund data and operations. The bank emphasises its efforts to “reform the trust funds framework” by “defining mobilisation objectives more strategically, simplifying and harmonizing agreements, improving cost recovery, and incorporating these funds more fully into the budgetary allocation process.”^41^ It points to the availability of financial and non-financial information about trust funds through AidFlows, the Financial Intermediary Fund Trustee website, and the World Bank Finances platform.^4^ ^15^ These resources, however, have major transparency problems for members of the public and researchers (fig 3). Figure 3 suggests specific ways in which these problems could be dealt with.

**Figure f3:**
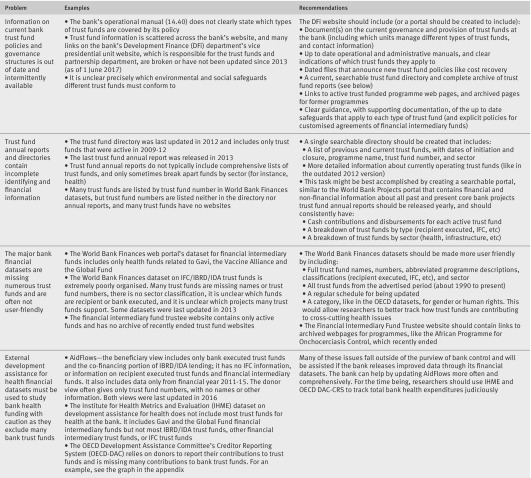
**Fig 3** Transparency issues and recommendations for bank health trust funds (IBRD/IDA and financial intermediary funds)

For a summary and in depth illustration of these drivers and potential perils of the bank’s health trust funds, see the case study of onchocerciasis programmes—funded by the oldest and longest running trust funds at the bank—in the appendix.

## Conclusion

The health financing landscape has transformed over the past 30 years as private aid flows increasingly overtake official development assistance.^42^ ^43^ With this shift, the supremacy of IBRD’s non-concessional financing model has ended, and IDA, IFC, and trust fund commitments now dwarf its own.^6^ At the same time, the bank has transformed from a country based lender to a development organisation with representation on the most global partnership programmes in the world.^16^

Trust funds reflect the World Bank’s eagerness to capitalise on these private aid flows for global health activities. This business decision largely explains the proliferation of ad hoc administrative and operational policies for IBRD/IDA trust funds and financial intermediary funds. Competitive interests of private investors to IFC also explain its restrictive access to information policy^44^ and customised policies about safeguards. In our view, however, trust funds have operated largely in the shadows and beyond the purview of members of the public, without having to conform to the measures taken to increase monitoring and accountability in the bank’s core work. We call on the bank to commit to its “Forward Look” strategy^41^ for a stronger World Bank Group by improving its trust fund transparency (fig 3 gives explicit recommendations) as a first step.

**Key messages**

Trust funds (non-core voluntary aid) for health projects made up nearly half of the World Bank’s total funding for health and social services in 2012-13
The Bank has four major types of trust funds: IBRD/IDA bank executed trust funds, IBRD/IDA recipient executed trust funds, financial intermediary funds, and IFC trust funds. These funds have distinct purposes, implementation mechanisms, and accountability frameworks
Benefits of the trust fund system for health include its potential for enhanced flexibility, capitalising on international momentum, measurable project outcomes, and investment in innovative areas or financing mechanisms
Risks of the Bank’s trust fund system for health include its potential for misaligned aid allocation, reduced Bank accountability, and inadequate transparency

## Web Extra

Extra material supplied by the author

## Notes

Contributors and sources: JW collected the data, analysed them, and drafted the initial version of the paper. DS helped to conceptualise and design the study and revised the draft. JW is a PhD student at the University of Edinburgh and her research focuses on onchocerciasis control programmes, defining success in global health, and the World Bank. DS holds a Wellcome Trust Investigator Award on the role of the World Bank in global health and is the coauthor of *Governing Global Health: Who Runs the World and Why?* (OUP, 2017). Data sources included World Bank reports, financial datasets, staff interviews, archived materials, and websites; external reports on multilateral financing; secondary literature on health policy; and archived materials at the WHO, Food and Agriculture Organization, and UN Development Programme.

Provenance and peer review: Commissioned; externally peer reviewed.
