# Supplementary material for: Earmarking for global health: benefits and perils of the World Bank’s trust fund model
Source: BMJ. 2017 Aug 31;358:j3394. doi: 10.1136/bmj.j3394 (PMC5594414; doi:10.1136/bmj.j3394)
Supplement: Supplementary file 1 — Appendix: supplementary material [file winj039789.ww1.pdf]

Appendix: Supplementary material [posted as supplied by author]

Case study of the onchocerciasis trust funds (1974-present)

Background – Onchocerciasis financing at the World Bank:

The Onchocerciasis Control Programme (OCP), initiated in partnership with the World Health Organization (WHO) in 1974, was the World Bank's first intercountry health programme and its first use of a trust fund in the health sector (4, 42). It began an international attack on onchocerciasis – a vector-borne tropical disease often resulting in permanent blindness – in eleven West African countries. The programme primarily funded vector control activities and, later, the identification, testing, and distribution of a new drug (ivermectin) for disease prevention. As both a donor to and trustee of the fund, IBRD took charge of marshalling donor investment in the programme (43). By 1995, the Onchocerciasis Control Programme investment was considered so successful that the bank and WHO launched a new trust fund programme, the African Programme for Onchocerciasis Control (APOC), to spread mass-drug administration of ivermectin to 20 additional African countries (44). In 2016, the WHO released the framework for a third programme for onchocerciasis control in Africa, the Expanded Special Programme for the Elimination of Neglected Tropical Diseases (ESPEN). The Expanded Special Programme relies on a complex trust fund mechanism to control onchocerciasis and four other neglected tropical diseases (45).

Benefits of the onchocerciasis funds for the World Bank and donors:

The onchocerciasis programmes illuminate the four drivers – flexibility, ability to capture international momentum, narrowly-defined goals and measurable outcomes, and utility in funding innovative activities – of the bank's health trust fund model:

- ***Flexibility*:** Mostly sovereign states and multilateral institutions contributed to the initial programme, the Onchocerciasis Control Programme (46). Following the success of its chemotherapy project in the 1980s, the flexibility of financial intermediary funds allowed Merck & Co. (a private corporation and developer of ivermectin) to enter as a donor. The African Programme for Onchocerciasis Control ultimately accepted funding from foundations, philanthropists, and the private sector, totaling a full 25% of its budget (47). The onchocerciasis funds further attracted donors because they retained the benefits of the bank as a secure multilateral financial institution. For example, records obtained from the bank archives indicate that some donors, particularly the United States, insisted that they would only be involved in the neglected tropical disease control programmes if their investments were given the security of a bank trusteeship (48). Additionally, the bank was able to retain many donors over nearly 40 years because the flexible trust fund structure allowed them to offer donors significant decision-making power, through representation on the programme's governing body (the Joint Programme Committee or Joint Action Forum, 42-43, 47).
- ***Harnessing international momentum*:** The first onchocerciasis trust fund was established following a series of high profile meetings on onchocerciasis (42). The programme was launched in spite of the relatively low burden of onchocerciasis compared to other infectious diseases and the fact that the bank had never been involved in a health project. Bank President Robert McNamara decided that the bank should become involved in a regional onchocerciasis problem, as a way to promote his poverty-alleviation agenda and emphasize the importance of health to international development (49-50). He quickly had the fund set-up and had the board of executive directors approve an IBRD donation of approximately 10% of annual programme expenses (51). Donors signed the first fund agreement in 1974, and funds were disbursed that same year, then on a quarterly basis each year, to the WHO for implementation (42-43, 47).
- ***Narrowly-defined goals and measurable outcomes*:** During the Onchocerciasis Control Programme, the WHO provided donors with clear numbers – such as the number of cases of blindness prevented and number of community distributors of ivermectin trained – that they could use to defend their investments (44, 52). While this funding was pooled without exceptions, the African Programme for Onchocerciasis Control gave donors more flexibility in earmarking their funds. For instance, Canada gave aid to all countries but had a specific preference on gender, and the United States directed funds to specific countries, including Mali, Senegal, and Ghana (53). The third iteration of the programme allows for even more earmarking, as pooled donor funds can be formally restricted to certain countries/regions or disease control activities (45). This allows donors to trace what their funding has bought at the country level.
- ***Fostering innovative projects and financing mechanisms:*** The Onchocerciasis Control Programme relied on unprecedented regional use of technologies, technical assistance, and research. Numerous aircrafts were required to spray insecticides in rivers both within countries and in border areas, as was research into more effective insecticides and drugs (42). These needs fell well outside of the capacity of any single African country's capabilities, but also did not match traditional bank investments, which in the 1970s did not include infectious disease control (49). A trust fund allowed the bank to begin a health sector project for the first time, and to pool funds for use across many countries.

Risks of the onchocerciasis funds for the World Bank and donors:

The onchocerciasis programmes also demonstrate three main concerns about the bank's use of voluntary funding for health – that trust funds allow donors to gain undue influence, that projects lack oversight and accountability, and that insufficient information is available to the public:

- ***Donor influence and verticalization:*** Archival sources indicate that the onchocerciasis programmes were beholden to donor pressures. Major donors were able to steer the Onchocerciasis Control Programme in the direction of chemotherapy research in the 1980s, and the programme invested significant resources visiting prospective donors and holding donor meetings (54-55). Furthermore, while the Onchocerciasis Control Programme is now hailed as an extremely successful public-private partnership (44, 52, 56), it spent more on vector control for onchocerciasis in the Upper Volta (Burkina Faso) alone than the entire Ministry of Health's budget during the late 1970s (57). The first two iterations of the programme have been criticized for beginning a trend of mass-drug administration, in which partnership programmes measure success through the number of drugs delivered for specific diseases (58). The third iteration is designed to avoid some of these criticisms of vertical programmes, by focusing on five diseases and technical assistance for health systems (45). Yet, its allowance of earmarking raises the risk that donors will provide funding to specific countries and diseases, irrespective of their disease burden and health systems status.
- ***Bank capacity and accountability erosion:*** As they are funded by financial intermediary funds, none of the onchocerciasis programmes are subject to traditional IBRD/IDA safeguards. Little information is publicly available about the customized agreements financial agreements made between donors and the bank for each trust fund, or about precisely which bank staff were involved in administering the funds. Additionally, the bank did not charge overhead or bill bank staff time during the African Programme for Onchocerciasis Control, in spite of the significant costs associated with trust fund negotiations, maintaining donor relations, and monitoring and reporting on the project (53). This may have "hollowed out" core staff capacity to contribute to other health and development projects.
- ***Transparency:*** Our attempt to track the disbursements of the first two iterations demonstrates the significant hurdles that continue to face external researchers studying trust funds (Figure 4). The graph below shows how tracking onchocerciasis funding through the major tools available to external researchers – official bank publications (Annual IBRD/IDA Reports, Trust Fund Reports, and peer-reviewed articles (59-61)), the World Bank Finances' financial intermediary fund dataset, the OECD-DAC multi-bi dataset (62), and AidFlows – yield radically different expenditure patterns. This limits researchers' ability to study the success of these programmes and their financing mechanisms.

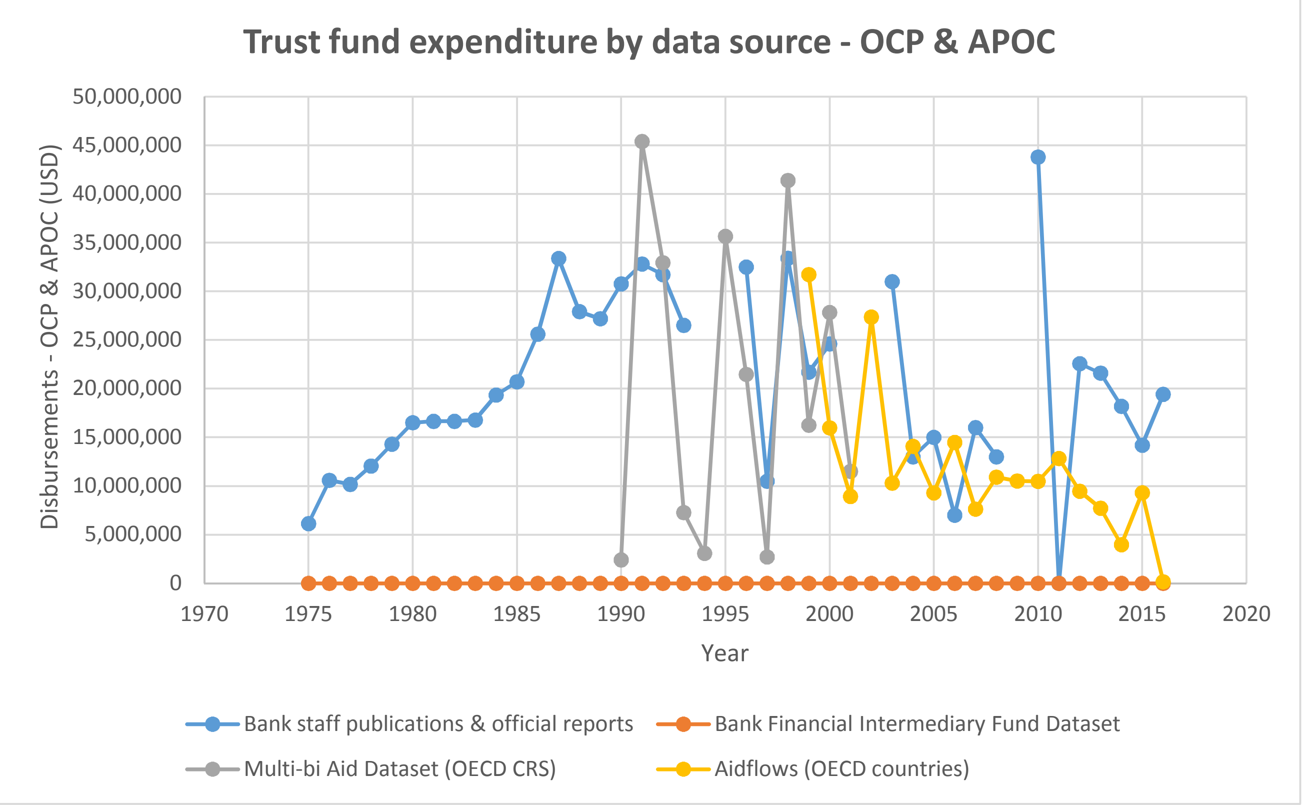

References:

1. Eichenauer V, Knack S. Poverty and poverty selectivity of World Bank trust funds – Policy Research Working Paper 7731 [Internet]. Washington DC: The World Bank Group; 2016 [cited 2017 Feb 6]. Available from: http://documents.worldbank.org/curated/en/495141467122731170/pdf/WPS7731.pdf
2. Reinsberg B. The implications of multi-bi financing for multilateral agencies: the example of the World Bank. In: Klingebiel S, Mahn T, Negre M, editors. The fragmentation of aid. Basingstoke: Palgrave Macmillan UK; 2016. p. 185-98.
3. Wagner L. How do earmarked funds change the geographical allocation of multilateral assistance? Working Paper 150 [Internet]. Paris: Foundation Pour Les Etudes et Recherches sur le Developpement International (FERDI); 2016 Mar 24 [cited 2017 Feb 6]. Available from: http://www.ferdi.fr/sites/www.ferdi.fr/files/evenements/presentations/seminaire_afd_-_presentation_l._wagner.pdf
4. Independent Evaluation Group. An evaluation of the World Bank's trust fund portfolio: trust fund support for development [Internet]. Washington DC: The World Bank Group; 2011 [cited 2017 Feb 6]. Available from: https://www.oecd.org/derec/worldbankgroup/48081370.pdf
5. World Bank Group. 2011 trust fund annual report [Internet]. Washington DC: The World Bank Group; 2011 [cited 2017 Feb 6]. Available from: http://documents.worldbank.org/curated/en/155091468314367064/2011-trust-fund-annual-report
6. Organisation for Economic Co-operation and Development. Multilateral aid 2015: better partnerships for a post-2015 world. Paris: OECD Publishing; 2015. doi: http://dx.doi.org/10.1787/9789264235212-en
7. Huq W. Analysis of recipient executed trust funds. CFP Working Paper Series 5 [Internet]. Washington DC: The World Bank Group; 2010 [cited 2017 June 5]. Available from: http://siteresources.worldbank.org/CFPEXT/Resources/CFP_Working_Paper_05.pdf
8. World Bank Group. 2013 trust fund annual report [Internet]. Washington DC: The World Bank Group; 2013 [cited 2017 Feb 6]. Available from: https://siteresources.worldbank.org/CFPEXT/Resources/299947-1274110249410/CFP_TFAR_AR13_High.pdf
9. World Bank. Trust funds at the World Bank – a guide for donors and partners [Internet]. Washington DC: The World Bank Group; 2009 [cited 2017 Feb 6]. Available from: http://siteresources.worldbank.org/EXTEXTINDTRAINI/Resources/Donor_Guide_11-03-09.pdf
10. World Bank Group. The World Bank Group modified cash basis trust funds: report on internal control over financial reporting and combined statements of receipts, disbursements and fund balance [Internet]. Washington DC: The World Bank Group; 2016 [cited 2017 Feb 6]. Available from: http://siteresources.worldbank.org/EXTABOUTUS/Resources/29707-1280852909811/FY16SingleAudit.pdf
11. World Bank. Financial intermediary funds: meeting global development challenges through international partnerships [Internet]. Washington DC: The World Bank; c2012 [cited 2017 Feb 6]. Available from: http://siteresources.worldbank.org/CFPEXT/Resources/299947-1267555827203/FIFBrochure_FINAL_Sep22.pdf
12. International Finance Corporation. IFC support to health public-private partnerships [Internet]. Washington DC: The World Bank; 2010 [cited 2017 June 5]. Available from: https://www.ifc.org/wps/wcm/connect/b10f4080498391e2865cd6336b93d75f/IFC_Support2Health_WEB.pdf?MOD=AJPERES&CACHEID=b10f4080498391e2865cd6336b93d75f
13. World Bank Group Finances. Paid in contributions to IBRD/IDA/IFC trust funds based on FY of receipt [Internet]. Washington DC: The World Bank Group [data accessed 2017 June 5]. Available from: https://finances.worldbank.org/Trust-Funds-and-FIFs/Paid-In-Contributions-to-IBRD-IDA-IFC-Trust-Funds-/nh5z-5qch
14. World Bank Group. Fact sheet on World Bank trust funds [Internet]. Washington DC: The World Bank Group; 2017 [cited 2017 June 5]. Available from: http://siteresources.worldbank.org/CFPEXT/Resources/TFfactsheetapril2017.pdf
15. Independent Evaluation Group. Opportunities and challenges from working in partnership: findings from IEG's work on partnership programs and trust funds. Washington DC: The World Bank Group; 2014. Available from: https://ieg.worldbankgroup.org/Data/reports/opps_and_challenges_from_partnership.pdf
16. World Bank Concessional Finance and Global Partnerships. Trust fund reforms: progress to date and future directions [Internet]. Washington DC: The World Bank Group; 2013 [cited 2017 Feb 6]. Available from: http://siteresources.worldbank.org/CFPEXT/Resources/299947-1396037011592/TFReform_SecM20130285_Public.pdf

17. World Bank. The World Bank Annual Report 2013 [Internet]. Washington DC: The World Bank; 2013 [cited 2017 Feb 6]. Available from: https://openknowledge.worldbank.org/bitstream/handle/10986/16091/9780821399378.pdf?sequence=1&isAllowed=y
18. Distler L. The third channel – new development aid financing in global health [dissertation]. Munich: Ludwig-Maximillans Universität; 2016. Available from: https://edoc.ub.uni-muenchen.de/19414/1/Distler_Layla.pdf
19. Smyth S, Triponel A. Funding Global Health. Health Hum Rights. 2013 June;15(1).
20. Independent Evaluation Group. Responding to global public bads: learning from evaluation of the World Bank experience with avian influenza 2006-13. Washington DC: The World Bank Group; 2014. Available from: http://ieg.worldbankgroup.org/Data/reports/avian_flu1.pdf
21. Sridhar D, Woods N. Trojan multilateralism: global cooperation in health. Global Policy. 2013 Nov 1;4(4):325-35.
22. World Bank. The World Bank operations manual: Bank procedures – trust funds [Internet]. Washington DC: World Bank; 2015 [cited 2017 June 5]. Available from: http://siteresources.worldbank.org/OPSMANUAL/112526-1124462138612/23702154/BP14.40-July2015.pdf
23. Storeng K. The GAVI Alliance and the 'Gates approach' to health system strengthening. Glob Public Health. 2014 9(8):865-879. doi: 10.1080/17441692.2014.940362
24. Bill & Melinda Gates Foundation. What we do [Internet]. Seattle WA: The Bill & Melinda Gates Foundation [cited 2017 June 5]. Available from: http://www.gatesfoundation.org/What-We-Do
25. Clinton C, Sridhar D. Governing global health: who runs the world and why? Oxford: Oxford University Press; 2017.
26. Browne S, Cordon R. Vertical funds: lessons for multilateralism and the UN [Internet]. New York: Future United Nations Development System, CUNY Graduate Center; 2015 [cited 2017 June 5]. Available from: https://www.futureun.org/media/archive1/briefings/FUNDS_Brief25_Jan2015_WHO_GAVI_GF.pdf
27. International Development Association. Project appraisal document on a proposed credit in the amount of SDR 50.2 million (US$74.68 million equivalent) to the Islamic Republic of Pakistan for a third partnership for polio eradication project [Internet]. Washington DC: World Bank; 2009 [cited 2017 June 5]. Available from: http://documents.worldbank.org/curated/en/379611468283490193/pdf/476740PAD0P114101Official0Use0Only1.pdf
28. World Bank, Oxford University. Disease control priorities in developing countries, second edition. Washington DC: International Bank for Reconstruction and Development; 2006. Available from: http://documents.worldbank.org/curated/en/632721468313531105/pdf/414600PAPER0Di101Official0Use0Only1.pdf
29. Reinsberg B, Michaelowa K, Knack S. Which donors, which funds? The choice of multilateral funds by bilateral donors at the World Bank. Policy Research Working Paper No. 7441 [Internet]. Washington DC: World Bank; 2015 [cited 2017 Feb 5]. Available from: https://openknowledge.worldbank.org/handle/10986/22872
30. Hafner T, Shiffman J. The emergence of global attention to health systems strengthening. Health Policy Plan. 2013 Jan 1;28(1):41-50.
31. Atun RA, Bennett S, Duran A. When do vertical (stand-alone) programmes have a place in health systems? [Internet]. Geneva: World Health Organization; 2008 [cited 2017 Feb 6]. Available from: http://www.who.int/management/district/services/WhenDoVerticalProgrammesPlaceHealthSystems.pdf
32. World Bank. Bank Directive – cost recovery framework for trust funds [Internet]. Washington DC: The World Bank; 2015 [cited 2017 Feb 6]. Available from: https://policies.worldbank.org/sites/ppf3/PPFDocuments/090224b082fe398d.pdf
33. World Bank. World Bank policies and procedures – operations manual [Internet]. Washington DC: World Bank [cited 2017 June 5]. Available from: https://policies.worldbank.org/sites/ppf3/Pages/Manuals/Operational%20Manual.aspx
34. World Bank. The World Bank environmental and social framework [Internet]. Washington DC: World Bank; 2017 [cited 2017 June 5]. Available from: http://documents.worldbank.org/curated/en/383011492423734099/pdf/114278-REVISED-Environmental-and-Social-Framework-Web.pdf
35. Reinsberg B. The bureaucratic politics of trust funds: evidence from the World Bank. Political Economy of International Organisations Conference 2016 Jan 7-9; Salt Lake City, Utah.

36. World Bank. Letter from Hartwig Schefer to Daniel Caitlin. Subject: World Bank support to Haiti [Internet]. Washington DC: World Bank; 2015 March 15 [cited 2017 June 5]. Available from: http://www.accountabilitycounsel.org/wp-content/uploads/2014/07/WB-Response-Letter-Haiti.pdf
37. Bretton Woods Project. World Bank support for mining expansion criticised [Internet]. London: United Kingdom; 2015 March 31 [cited 2017 June 5]. Available from: http://www.brettonwoodsproject.org/2015/03/world-bank-support-for-mining-expansion-criticised/
38. World Bank Group. Forward look – a vision for the World Bank Group in 2030 [Internet]. Washington DC: World Bank Group; 2016 Sept 20 [cited 2017 June 5]. Available from: http://siteresources.worldbank.org/DEVCOMMINT/Documentation/23732171/DC2016-0008.pdf
39. International Finance Corporation. The business of health in Africa: partnering with the private sector to improve people's lives [Internet]. Washington DC: The World Bank Group; 2008 [cited 2017 June 5]. Available from: http://documents.worldbank.org/curated/en/878891468002994639/The-business-of-health-in-Africa-partnering-with-the-private-sector-to-improve-peoples-lives
40. Shiffman J. Knowledge, moral claims and the exercise of power in global health. Int J Health Policy Manag. 2014 Nov 14;3:297-299. doi: 10.15171/ijhpm.2014.120
41. International Finance Corporation. Access to information policy [Internet]. Washington DC: The World Bank Group; 2012 Jan 1 [cited 2017 June 5]. Available from: http://www.ifc.org/wps/wcm/connect/98d8ae004997936f9b7bffb2b4b33c15/IFCPolicyDisclosureInformation.pdf?MOD=AJPERES
42. Samba E. The Onchocerciasis Control Programme in West Africa: an example of effective public health management. Geneva: World Health Organization; 1994.
43. World Health Organization. Success in Africa: the Onchocerciasis Control Programme in West Africa, 1972-2002. Geneva: World Health Organization; 2002.
44. Independent Evaluation Group. The World Bank's involvement in global and regional partnership programs [Internet]. Washington DC: The World Bank Group; 2011 [cited 2017 Feb 6]. Available from: https://www.oecd.org/derec/worldbankgroup/48296274.pdf
45. WHO Regional Office for Africa NTD Programme. Framework for the establishment of the Expanded Special Project for Elimination of Neglected Tropical Diseases [Internet]. Brazzaville: The World Health Organization; 2015 [cited 2017 Feb 6]. Available from: http://www.afro.who.int/espen
46. United States Agency for International Development. Draft project paper, Onchocercaisis Control Program Phase IV [Internet]. Washington DC: USAID; c1992. Available from: http://pdf.usaid.gov/pdf_docs/pdabn465.pdf
47. Bundy D, Dhomun B, Daney X, Schultz L, Tembon A. Investing in onchocerciasis control: financial management of the African Programme for Onchocerciasis Control (APOC). Plos Negl Trop Dis. 2015 May 14;9(5):e0003508. doi: 10.1371/journal.pntd.0003508
48. Office Memorandum, Arlene Fonaroff to John Evans, November 7, 1979; Background Materials for Bank Participation in Tropical Disease Research; WB IBRD/IDA 89 Records of the Population, Health and Nutrition Sector; 1103166, World Bank Group Archives, Washington, D.C., United States.
49. Fair M. World Bank HNP Timeline [Internet]. Washington DC: World Bank; c2008 [cited 2017 June 5]. Available from: http://siteresources.worldbank.org/EXTWBASSHEANUTPOP/Resources/timeline.pdf
50. Bump J. The lion's gaze: African river blindness from tropical curiosity to international development [dissertation]. Baltimore MD: Johns Hopkins University; 2004.
51. The World Bank Group Archives Oral History Program. Transcript from an interview with Bruce Benton, May 19 and 16, June 13 and 27, 2006, Washington DC, Interview by Probert P. Grathwol [Internet]. Washington DC: World Bank Group; c2006 [cited 2017 June 5]. Available from: http://documents.worldbank.org/curated/en/599511474565572245/pdf/108395-TRANSCRIPT-PUBLIC-Bruce-Benton-redacted.pdf
52. World Bank. A successful public-private partnership model [Internet]. Washington DC: World Bank Group; 2014 [cited 2017 Feb 6]. Available from: http://documents.worldbank.org/curated/en/130941468000273221/A-successful-public-private-partnership-model
53. Thanks to Bernhard Reinsberg from sharing his 2013 interview material with a key official (World Bank financial manager) from the African Programme for Onchocerciasis Control trust fund.
54. Letter from A.W. Clausen to Halfdan Mahler, June 25, 1984; Correspondence – Volume 1; WB IBRD/IDA Clausen Papers – World Health Organization; 1633229, World Bank Group Archives, Washington, D.C., United States
55. Office Memorandum, Stephen Denning to Bilseh Alisbah, May 26, 1982; Onchocerciasis Control Program: Chemotherapy Project; WB IBRD/IDA 89 Records of the Population, Health and Nutrition Sector; 1300677, World Bank Group Archives, Washington, D.C., United States.

56. Bush S, Hopkins A. Public–private partnerships in neglected tropical disease control: the role of nongovernmental organisations. Acta tropica. 2011 Sep 30;120:S169-72.
57. Notes for the Record, Task Force Meeting, 1979 July 23; UN 17-16, Onchocerciasis Control Programme, July – August 1979; Food and Agriculture Organization Archives, Rome.
58. Parker M, Allen T. Does mass drug administration for the integrated treatment of neglected tropical disease really work? Assessing evidence for the control of schistosomiasis and soil-transmitted helminths in Uganda. Health Res Policy Syst. 2011 Jan 9;9(1):3. doi: 10.1186/1478-4505-9-3
59. Prost A, Prescott N. Cost-effectiveness of blindness prevention by the Onchocerciasis Control Programme in Upper Volta. Bull World Health Organ. 1984;62(5):795.
60. Kim A, Benton B. Cost-benefit analysis of the Onchocerciasis Control Program (OCP), World Bank Technical Paper Number 282. Washington DC: The World Bank; 1995.
61. World Bank Concessional Finance and Global Partnerships Vice Presidency. Directory of programs supported by trust funds, as of March 21, 2012 [Internet]. Washington DC: The World Bank Group; c2012 [cited 2017 Feb 6]. Available from: http://siteresources.worldbank.org/CFPEXT/Resources/299947-1274110249410/DirectoryofPrograms_TrustFunds_2012.pdf
62. Eichenauer V, Reinsberg B. What determines earmarked funding to international development organizations? Evidence from the new multi-bi aid dataset. Rev Int Organ. 2017 Jan 26. doi: 10.1007/s11558-017-9267-2
